# Correlations Between the Histologic Growth Patterns of Peritoneal and Liver Metastases From Colorectal Cancer

**DOI:** 10.1245/s10434-026-20005-8

**Published:** 2026-06-29

**Authors:** Noémie Zenaty, Radu Bachmann, Charif Khaled, Pieter Demetter, Francesco Sclafani, Pamela Baldin, Ana Veron Sanchez, Michel Moreau, Vincent Donckier, Gabriel Liberale

**Affiliations:** 1 Department of Surgical Oncology, Université Libre de Bruxelles (ULB), The Brussels University Hospital (H.U.B), Jules Bordet Institute, Brussels, Belgium; 2 Department of Surgery, Cliniques Universitaires Saint-Luc (UCLouvain), Brussels, Belgium; 3 Department of Pathology, Laboratory for Experimental Gastroenterology, Université Libre de Bruxelles, Brussels, Belgium; 4 Department of Gastrointestinal Oncology, Université Libre de Bruxelles (ULB), The Brussels University Hospital (H.U.B), Jules Bordet Institute, Brussels, Belgium; 5 Department of Pathology, Cliniques Universitaires Saint-Luc (UCLouvain), Brussels, Belgium; 6 Department of Radiology, Université Libre de Bruxelles (ULB), The Brussels University Hospital (H.U.B), Jules Bordet Institute, Brussels, Belgium; 7 Data Centre and Statistics Department, Université Libre de Bruxelles (ULB), The Brussels University Hospital (H.U.B), Jules Bordet Institute, Brussels, Belgium

**Keywords:** Histological growth pattern, Colorectal cancer, Peritoneal metastasis, Liver metastasis, Cytoreductive surgery, Liver resection

## Abstract

**Background:**

Histologic growth patterns (HGPs) of both peritoneal metastasis (PM) and liver metastasis (LM) have emerged as prognostic factors for patients undergoing curative surgery for metastatic colorectal cancer (CRC). The main objectives of the study were, first, to evaluate the correlation between PMHGP and LMHGP of CRC patients who underwent curative-intent cytoreductive surgery (CRS) and LM resection and, second, to assess the prognostic impact of HGP type.

**Methods:**

This retrospective bicentric study included patients treated with both CRS and LM resection for metastatic CRC. Histologic growth patterns were classified as pushing (P-HGP) and infiltrating (I-HGP) for PM, and as desmoplastic HGP (D-HGP) and non-desmoplastic (ND-HGP) for LM. Correlation between PMHGP and LMHGP was assessed using the chi-square test. Overall survival (OS) and disease-free survival (DFS) were evaluated using Kaplan-Meier and Cox regression.

**Results:**

The study included 44 patients. Of the 44 patients, 9 (20.5%) had P-HGP + D-HGP, 23 (52.3%) had I-HGP + ND-HGP, 5 (11.4%) had P-HGP + ND-HGP, and 7 (15.9%) had I-HGP + D-HGP. Significant correlation was observed between PMHGP and LMHGP (*p* = 0.0085; Φ = 0.3965). The patients with I-HGP + ND-HGP had shorter OS and DFS than those with other combinations: 34 versus 64 months (HR, 2.067; *p* = 0.0672) for OS, and 14 versus 25 months (hazard ratio [HR], 1.775; *p* = 0.1071) for DFS.

**Conclusions:**

This study highlighted a significant correlation between PMHGP and LMHGP in metastatic CRC. However, survival trends were nonsignificant.

**Supplementary Information:**

The online version contains supplementary material available at https://doi.org/10.1245/s10434-026-20005-8.

Colorectal cancer (CRC) is the second leading cause of cancer-related death worldwide.^1^ At diagnosis, approximately 7% of patients present with synchronous peritoneal metastasis (PM) and 15% with synchronous liver metastases (LM).^2–4^ Furthermore, approximately 19% of patients experience metachronous PM, and 16 to 20% of patients experience metachronous LM, even after initial curative-intent surgery.^2,4,5^

For patients with limited extent PMCRC, macroscopic complete cytoreductive surgery (CRS) with perioperative chemotherapy remains the only curative option, with a reported median overall survival (OS) of 41.2 months and a 5-year survival rate ranging from 22 to 50.5%.^6,7^ Patients with LMCRC usually are treated with liver resection ± radiofrequency ablation, which is associated with a median OS of 43.2 months and a 5-year survival rate ranging from 15 to 67%.^8,9^ A recent study reported a median OS of 47.6 months and a 5-year survival rate of 39% for CRC patients with synchronous PM and LM who undergo combined CRS with hyperthermic intraperitoneal chemotherapy (HIPEC) and liver resection.^10^

Unfortunately, the lack of accurate prognostic markers for patients who are candidates for surgery makes the outcomes for these patients difficult to predict, with many having rapid postoperative recurrence, rendering surgery futile. For both PM and LM, the characteristics of the primary tumor and the metastatic lesions have been studied in an effort to predict the outcome of CRC patients undergoing surgery.^11^ The main prognostic factors for PM include the completeness of cytoreduction (CC) and the extent of peritoneal disease assessed by the peritoneal cancer index (PCI).^12^ For LM patients, the studies have included the size (>5 cm) and number (≥2) of LMs, node-positive primary tumor, preoperative carcinoembryonic antigen [CEA] level (>200 ng/ml), and disease-free interval shorter than 12 months between the diagnosis of the primary tumor and the occurrence of LM.^5,13^

In the last few decades, additional prognostic factors have been explored and identified in patients with PMCRC and LMCRC, including tumor molecular profiles and clinical biomarkers.^14–17^ Several studies have highlighted the value of morphologic markers such as the histologic growth pattern (HGP) in LMCRC,^18–24^ and more recently, in PMCRC.^25,26^ These patterns represent different types of interactions between metastatic tumor cells and the stromal or parenchymal cells of the invaded organ.^27^ Findings have shown that HGPs carry an independent prognostic value for both OS and disease-free survival (DFS).^24,26^

Several distinct patterns have been identified. In the peritoneum, two patterns have been described: a pushing type associated with a better prognosis (P-HGP) and an infiltrating type (I-HGP) correlated with worse oncologic outcomes (OS: 60 vs 39 months; *p* = 0.014).^26^ In the liver, findings have shown the desmoplastic HGP (D-HGP) to be associated with favorable outcomes, whereas non-desmoplastic HGP (ND-HGP), including both replacement (R-HGP) and pushing (P-HGP) patterns, have been linked to a poorer prognosis (OS: 72 vs. 49 and 24 months, respectively; *p* < 0.001).^24^

Theoretically, each metastatic lesion may express multiple coexisting HGPs in varying proportions. However, most studies have reported minimal heterogeneity in HGP proportions, both within individual PMs or LMs (intra-metastatic) and between different nodules located in the same metastatic site (inter-metastatic) from the same patient.^25–28^

To date, no study to our knowledge has compared the HGP of PM and LM in the same cohort. Determining whether a correlation exists between these two anatomic sites and whether this correlation carries prognostic implications for oncologic outcomes could provide new insights for a more accurate estimation of OS and DFS after curative-intent surgery of metastatic CRC. Such findings may ultimately support the development of more personalized treatment strategies based on the HGP profile of each patient with combined PM and LM, potentially improving both surgical decision-making and overall therapeutic management.

This study aimed to evaluate correlations between the HGP of PM and LM in patients undergoing curative-intent CRS and LM resection for PM and LMCRC, and to assess the prognostic value of HGP for this population.

## Patients and Methods

### Design, Patient Selection, and Data Collection

This retrospective, bicentric study was conducted in collaboration with Jules Bordet Institute, The Brussels University Hospital (H.U.B), Université Libre de Bruxelles (ULB), and Cliniques Universitaires Saint-Luc (UCLouvain). Ethical approval was obtained from the ethics committee at each institution (CE3900 and 2023/20AOU/387). The study included patients who underwent complete CRS (CC-0) ± HIPEC (intraperitoneal oxaliplatin 460 mg/m^2^ at 41–43 °C for 30 min and systemic 5-fluorouracil), and curative-intent LM resection for CRC between January 2010 and December 2023. The study excluded patients with unavailable or non-informative histologic slides and patients with a PCI higher than 20 or macroscopically incomplete CRS (CC-1/CC-2).

Clinicopathologic data were collected for each patient, pseudonymized, and entered in our institution’s REDCap database. These data included demographic characteristics, details of the primary CRC (neoadjuvant and/or adjuvant chemotherapy, preoperative CEA, histopathology, grade, mismatch repair (MMR) phenotype, and KRAS/BRAF mutations), specific features of PM and LM (number, size, and chronology of metastases), and surgical information (PCI, CC, and HIPEC). The study classified LM and PM as synchronous if diagnosed within 12 months after the diagnosis of the primary tumor, and as metachronous if identified beyond that period.

### Histopathologic Analysis: Characterization of PMCRC and LMCRC HGP

All pathology reports of eligible patients were reviewed. For each patient, up to three of the largest LM and peritoneal nodules were selected. The corresponding hematoxylin and eosin (H&E)-stained slides showing the tumor-organ interface were analyzed using a Leica bright-field microscope (× 10 objective) by two expert pathologists (P.D., P.B., one from each institution) blinded to clinical data. Slides with the largest circumferential margins between the tumor and surrounding peritoneal or hepatic tissues were included.

According to the guidelines, results were recorded as average HGP scores, representing either the mean percentage of each HGP per metastasis (if multiple slides were available) and/or per patient (in case of multiple metastases). A pattern was considered dominant if ≥ 51% of the tumor-organ interface exhibited that pattern.^24,26^

Two distinct PMHGPs were identified as follows: P-HGP characterized by a fibrous rim pushing back the healthy peritoneal tissue with no focal tumor infiltration into the surrounding peritoneal lining and I-HGP, which presents focal penetration of tumor cells into the peritoneal lining without a separating fibrous rim.^25,26^ Similarly, three distinct LM HGPs were assessed according to international consensus scoring guidelines, updated in 2022.^24^ As previous studies have shown that the most discriminative prognostic categorization is obtained by comparing patients who have a dominant D-HGP with those who do not, the patients were classified as D-HGP or ND-HGP (grouping replacement-HGP and P-HGP).^24^

### Statistical Analysis

Data were analyzed by our statistician (M.M.) using STATA software (StataCorp, College Station, TX, USA). Descriptive analysis for categorical variables is reported as frequencies and percentages, whereas continuous variables are expressed as mean ± standard deviation (SD) or median ± interquartile range (IQR). Associations between PMHGP and LMHGP were evaluated using the chi-square test.

Survival estimates were obtained using the Kaplan-Meier method and compared with the log-rank test. Factors associated with survival were evaluated through uni- and multivariate Cox regression, with results expressed as hazard ratios (HRs) and 95% confidence intervals (95% CIs). Overall survival was defined as the time from diagnosis of PM or LM to death or last follow-up visit, and DFS from first surgery (CRS and/or liver resection) to non-curatively treated relapse or death. Patients without events were censored at the last follow-up visit. A *p* value lower than 0.05 was considered statistically significant.

## Results

A total of 92 patients underwent CRS ± HIPEC for PMCRC and had associated LM. Among these patients, 48 were excluded for the following reasons: 23 patients for having non-surgical management of LM, 22 patients for unavailable/non-contributing slides, 2 patients for PCI higher than 20, and 1 patient for having undergone incomplete CRS (CC-1). The final study population was 44 patients.

Patient and disease characteristics are summarized in Table 1.

**Table 1:** Clinicopathologic characteristics of the global population and comparisons between patients with different pattern combinations

Variables	Population (*n* = 44) *n* (%)	P-HGP + D-HGP (*n* = 9) *n* (%)	P-HGP + ND-HGP or I-HGP + D-HGP (*n* = 12) *n* (%)	I-HGP + ND-HGP (*n* = 23) *n* (%)	*p* Value
Sex					0.5122
M	17 (38.6)	4 (44.4)	6 (50.0)	7 (30.4)	
F	27 (61.4)	5 (55.6)	6 (50.0)	16 (69.6)	
Age (years)					1.0000
≤58	21 (47.7)	4 (44.4)	6 (50.0)	11 (47.8)	
>58	23 (52.3)	5 (55.6)	6 (50.0)	12 (52.2)	
ASA					0.4317
1–2	31 (70.5)	8 (88.9)	9 (75.0)	14 (60.9)	
3	13 (29.5)	1 (11.1)	3 (25.0)	9 (39.1)	
Site					1.0000
Colon	40 (90.9)	8 (88.9)	11 (91.7)	21 (91.3)	
Rectum	4 (9.1)	1 (11.1)	1 (8.3)	2 (8.7)	
T					0.2021
3	19 (43.2)	3 (33.3)	3 (25.0)	13 (56.5)	
4	25 (56.8)	6 (66.7)	9 (75.0)	10 (43.5)	
N					0.0550
0	8 (18.2)	1 (11.1)	2 (16.7)	5 (21.7)	
1	15 (34.1)	0 (0.0)	6 (50.0)	9 (39.1)	
2	21 (47.7)	8 (88.9)	4 (33.3)	9 (39.1)	
Grade					0.7822
Low	9 (20.5)	1 (11.1)	3 (25.0)	5 (21.7)	
Moderate	33 (75.0)	7 (77.8)	9 (75.0)	17 (73.9)	
High	2 (4.5)	1 (11.1)	0 (0.0)	1 (4.3)	
Mucinous					0.1593
No	37 (84.1)	8 (88.9)	8 (66.7)	21 (91.3)	
Yes	7 (15.9)	1 (11.1)	4 (33.3)	2 (8.7)	
MMR (missing: 7)					0.1892
MSS	36 (97.3)	6 (85.7)	11 (100.0)	19 (100.0)	
MSI	1 (2.7)	1 (14.3)	0 (0.0)	0 (0.0)	
KRAS (missing: 12)					0.5692
Wild	14 (43.8)	4 (66.7)	4 (40.0)	6 (37.5)	
Mutated	18 (56.3)	2 (33.3)	6 (60.0)	10 (62.5)	
BRAF (missing: 19)					0.4800
Wild	24 (96.0)	5 (20.0)	6 (24.0)	13 (52.0)	
Mutated	1 (4.0)	1 (4.0)	0 (0.0)	0 (0.0)	
Preoperative chemotherapy					0.0825
Before both	19 (43.2)	3 (33.3)	5 (41.7)	11 (47.8)	
Only before CRS	5 (11.3)	0 (0.0)	1 (8.3)	4 (17.4)	
Only before LM resection	8 (18.2)	4 (44.5)	3 (25.0)	1 (4.3)	
None	12 (27.3)	2 (22.2)	3 (25.0)	7 (30.4)	

*HGP* Histological growth pattern, *P-HGP* Pushing HGP, *D-HGP* Desmoplastic HGP, *ND-HGP* Non-desmoplastic HGP, *I-HGP* Infiltrating HGP, *ASA* American Society of Anesthesiologists, *MMR* Mismatch repair, *MSS* Microsatellite stable, *MSI* Microsatellite instability, *CRS* Cytoreductive surgery, *LM* Liver metastases

The study included 17 males and 27 females with a median age of 58 years. The primary tumor originated from the colon in 40 patients (90.9%) and the rectum in 4 patients (9.1%). With regard to preoperative treatments, 19 patients (43.2%) received chemotherapy before both CRS and LM resection, 5 patients (11.3%) received chemotherapy only before CRS, 8 patients (18.2%) received chemotherapy only before LM resection, and 12 patients (27.3%) did not receive preoperative chemotherapy at all. The clinicopathologic characteristics of the patients were similar between the two participating centers (IJB and CUSL) (Supplementary File 1).

Metastasis-specific characteristics are presented in Table 2. Of the 44 patients, 15 presented with PM first (34.1%), 16 with LM first (36.4%), and 13 with simultaneous PM and LM (29.5%). In 19 patients (43.2%) PM was synchronous, and in 25 patients (56.8%), PM was metachronous. The mean PCI for the entire cohort was 9.5, and 34 patients (77.3%) received HIPEC during CRS. Regarding LM, 24 patients (54.5%) had synchronous LM, and 20 patients (45.5%) had metachronous LM. The mean number of LMs on preoperative imaging was 2.4.

**Table 2:** Metastases-specific characteristics of the global population and comparisons between patients with different pattern combinations

	Variables	Population (*n* = 44) *n* (%)	P-HGP + D-HGP (*n* = 9) *n* (%)	P-HGP + ND-HGP or I-HGP + D-HGP (*n* = 12) *n* (%)	I-HGP + ND-HGP (*n* = 23) *n* (%)	*p* Value
PM	Chronology of PM					0.3705
	Synchronous carcinosis	19 (43.2)	2 (22.2)	5 (41.7)	11 (47.8)	
	Metachronous carcinosis	25 (56.8)	7 (77.8)	7 (58.3)	12 (52.2)	
	PCI					**0.0040**
	≤9.5	26 (59.1)	9 (100.0)	8 (66.7)	9 (39.1)	
	>9.5	18 (40.9)	0 (0.0)	4 (33.3)	14 (60.9)	
	CEA (ng/mL) (missing: 4)					1.0000
	≤5	24 (60.0)	5 (55.6)	6 (60.0)	13 (61.9)	
	>5	16 (40.0)	4 (44.4)	4 (40.0)	8 (38.1)	
	Completeness of CRS					—
	CC-0	44 (100.0)	9 (100.0)	12 (100.0)	23 (100.0)	
	HIPEC					0.3915
	No	10 (22.7)	2 (22.2)	1 (8.3)	7 (30.4)	
	Yes	34 (77.3)	7 (77.8)	11 (91.7)	16 (69.6)	
	Nb of peritoneal nodules analyzed					—
	Mean ± SD	1.9 ± 0.78	1.2 ± 0.44	2.1 ± 0.79	2.0 ± 0.77	
	Nodule size (cm)					0.5484
	Mean ± SD	1.6 ± 0.83	1.7 ± 1.19	1.8 ± 0.92	1.5 ± 0.69	
	PM HGP					**<0.0001**
	P-HGP ≥51%	14 (31.8)	9 (100.0)	5 (41.7)	0 (0.0)	
	I-HGP ≥51%	30 (68.2)	0 (0.0)	7 (58.3)	23 (100.0)	
LM	Chronology of LM					0.1921
	Synchronous	24 (54.5)	5 (55.6)	9 (75.0)	10 (43.5)	
	Metachronous	20 (45.5)	4 (44.4)	3 (25.0)	13 (56.5)	
	Nb of LM on preop imaging					0.1104
	Mean ± SD	2.4 ± 1.59	2.8 ± 1.64	3.0 ± 1.65	1.9 ± 1.44	
	Nb of LM analyzed					—
	Mean ± SD	1.4 ± 0.73	1.0 ± 0.00	1.8 ± 0.94	1.3 ± 0.65	
	Liver metastases HGP					**<0.0001**
	D-HGP ≥51%	16 (36.4)	9 (100.0)	7 (58.3)	0 (0.0)	
	ND-HGP ≥51%	28 (63.6)	0 (0.0)	5 (41.7)	23 (100.0)	
PM + LM	First metastasis treated with curative intent					0.0544
	Peritoneum	15 (34.1)	0 (0.0)	3 (25.0)	12 (52.2)	
	Liver	16 (36.4)	5 (55.6)	5 (41.7)	6 (26.1)	
	Peritoneum + liver	13 (29.5)	4 (44.4)	4 (33.3)	5 (21.7)	

Significant *p* <0.05

*HGP* Histological growth pattern, *P-HGP* Pushing HGP, *D-HGP* Desmoplastic HGP, *ND-HGP* Non-desmoplastic HGP, *I-HGP* Infiltrating HGP, *PM* Peritoneal metastases, *PCI* Peritoneal cancer index, *CEA* Carcinoembryonic antigen, *CRS* Cytoreductive surgery, *CC* Completeness of cytoreduction, *HIPEC* Hyperthermic intraperitoneal chemotherapy, *SD* Standard deviation, *LM* Liver metastases

### Analysis of the HGP

From the 44 patients, 83 PMs and 62 LMs were analyzed for HGP. A mean of 1.9 PMs and 1.4 LMs were analyzed per patient. The mean PM size was 1.6 cm. Among the 338 slides reviewed, 279 were deemed informative. Full agreement on the nature of the dominant HGP was reached between pathologists in 100% of cases, with minor discrepancies (≤15%) for only four slides, which did not affect the final classifications.

**Fig. 1:**
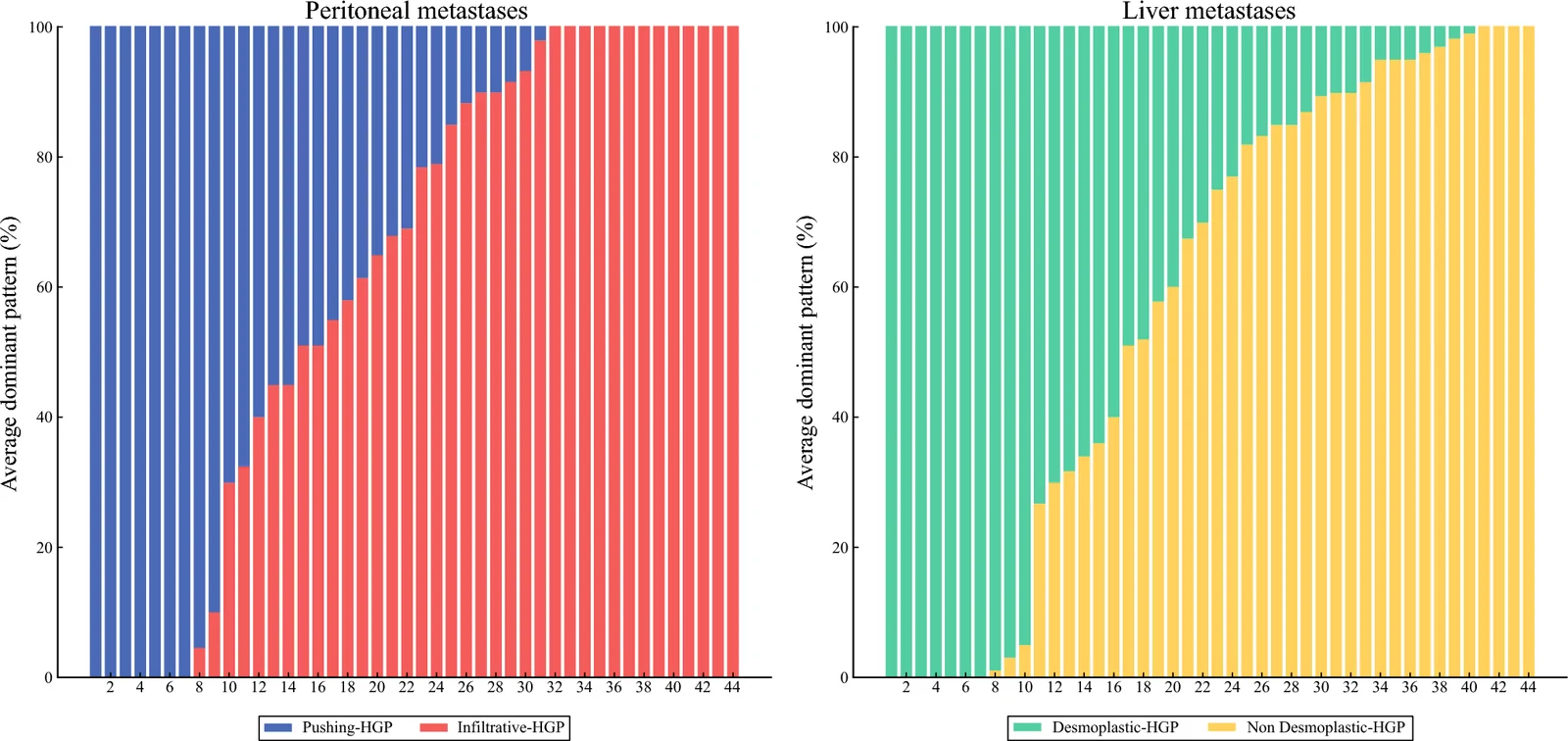
Proportions of histologic growth patterns (HGPs) in individual patients.

For PM, 14 patients (31.8%) had dominant P-HGP, and 30 patients (68.2%) exhibited dominant I-HGP. Pure patterns were observed in 7 patients (15.9%) for P-HGP and in 13 patients (29.5%) for I-HGP, whereas 24 patients (54.6%) exhibited mixed patterns. Similarly for LM, a dominant D-HGP was observed in 16 patients (36.4%), and a dominant ND-HGP was identified in 28 patients (63.6%). Pure patterns were identified in 7 patients (15.9%) for D-HGP and 4 patients (9.1%) for ND-HGP, whereas 33 patients (75.0%) demonstrated mixed patterns (Fig. 1).

Furthermore, minimal variations in HGP proportions were observed in cases with multiple slides per metastasis and/or multiple metastases per patient. Intra-nodular discordance of the dominant HGP was defined as discrepancies between multiple slides from the same nodule. This was observed in one PM in 4 patients (9.1%) and in one LM in 4 patients (9.1%). Inter-nodular discordance was defined as a difference in the dominant HGP between at least two nodules from the same patient. This was observed among PMs of four patients (9.1%) and among LMs of three patients (6.8%).

### Correlations Between HGP of PMCRC and LMCRC

Table 3 shows the correlation between the dominant HGP for PM and LM. The patients were categorized into four groups based on the different combinations of HGP as follows: 9 patients (20.4%) had P-HGP + D-HGP, 23 patients (52.3%) had I-HGP + ND-HGP, 5 patients (11.4%) had P-HGP + ND-HGP, and 7 patients (15.9%) had I-HGP + D-HGP. A significant correlation was observed (*p* = 0.0085) for the HGP of a good prognosis (P-HGP and D-HGP) and for the HGP of a bad prognosis (I-HGP and ND-HGP), with a moderate association strength (phi coefficient Φ = 0.3965).

**Table 3:** Correlations between histologic growth patterns of peritoneal and liver metastases

HGP		PM		Total *n* (%)	*p* Value (chi^2^ test)	Φ *(*phi coefficient*)*
		P-HGP ≥51% *n* (%)	I-HGP ≥51% *n* (%)			
LM	D-HGP ≥51%	9 (20.45)	7 (15.91)	16 (36.36)	**0.0085**	**0.3965**
	ND-HGP ≥51%	5 (11.37)	23 (52.27)	28 (63.64)		
TOTAL		14 (31.82)	30 (68.18)	44 (100)		

Significant *p* <0.05

*PM* Peritoneal metastases, *HGP* Histologic growth pattern, *P-HGP* Pushing HGP, *I-HGP* Infiltrating HGP, *LM* Liver metastases, *D-HGP* Desmoplastic HGP, *ND-HGP* Non-desmoplastic HGP

### Correlations Between Clinicopathologic Characteristics and HGP Combinations

The different pattern combinations were significantly correlated with PCI (*p* = 0.004), but not with other evaluated factors, including patient demographics, primary tumor and metastases characteristics, and use of preoperative chemotherapy. The combination of P-HGP + D-HGP was associated with a significantly lower PCI than the mixed group (P-HGP + ND-HGP and I-HGP + D-HGP), and both had a significantly lower PCI than the combination of I-HGP + ND-HGP (Table 2). Moreover, a statistical trend was observed between the pattern combination and the location of the first metastasis treated with curative intent (*p* = 0.0544). In the favorable HGP group (P-HGP + D-HGP), 55.6% of the patients had LM first, whereas in the unfavorable HGP group (I-HGP + ND-HGP) 52.2% had PM first.

### Prognostic Value of Histologic Growth Pattern

**Fig. 2:**
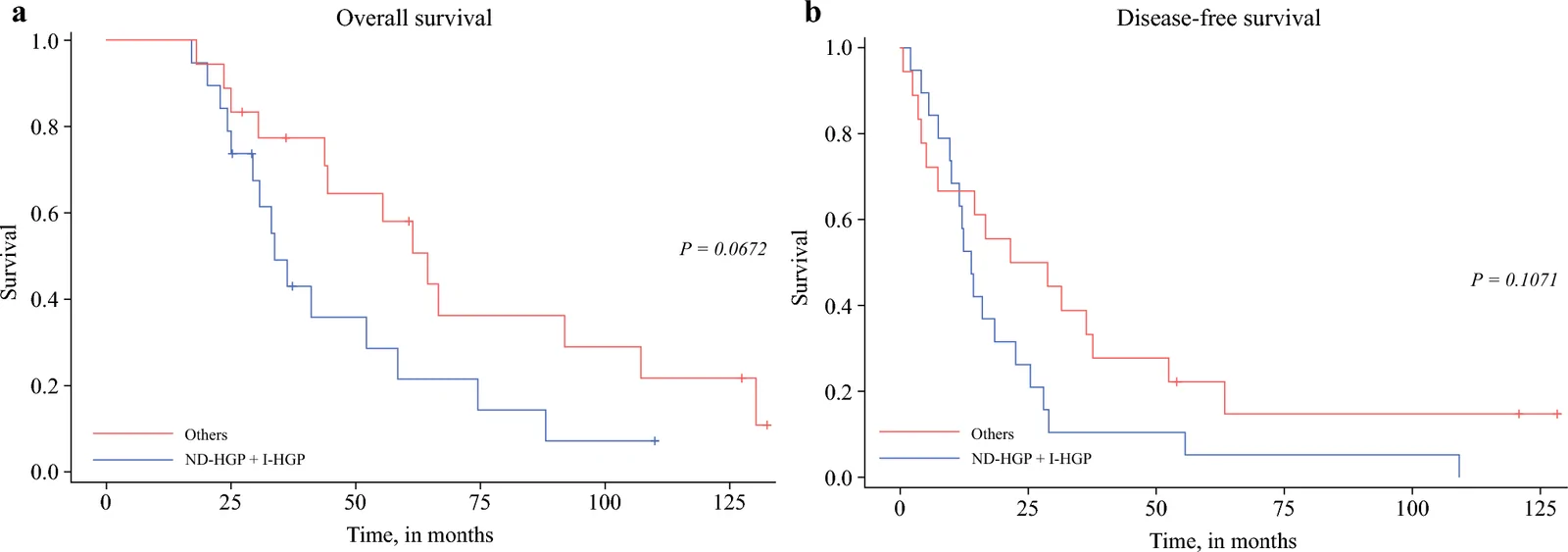
Kaplan-Meier survival plots of (**a**) OS and (**b**) DFS stratified by histologic growth pattern combinations: ND-HGP + I-HGP vs others (D-HGP + P-HGP or ND-HGP + P-HGP or D-HGP + I-HGP). OS, overall survival; DFS, disease-free survival; D-HGP, desmoplastic HGP; P-HGP, pushing HGP; ND-HGP, non-desmoplastic HGP; I-HGP, infiltrating HGP

The median follow-up period was 2 years. The median OS was 52 months (95% CI, 33–64 months), and the median DFS was 16 months (95% CI, 11–28 months). Among the different HGP-based stratifications tested, comparison between the I-HGP + ND-HGP group and all other combinations (P-HGP + D-HGP, P-HGP + ND-HGP, and I-HGP + D-HGP) showed the clearest survival trend (Table 4). In univariate Cox analysis, the patients with dominant I-HGP + ND-HGP had a shorter OS (34 vs 64 months; HR, 2.067; 95% CI, 0.950–4.499; *p* = 0.0672) and DFS (14 vs 25 months; HR, 1.775; 95% CI, 0.883–3.565; *p* = 0.1071), although the differences were not statistically significant. Survival curves visually support this trend, but the crossing of DFS curves suggest a potential violation of the proportional hazards assumption, warranting formal testing (Fig. 2).

**Table 4:** Univariate analysis of the prognostic impact of histologic growth pattern combinations from peritoneal metastasis and liver metastases

	Overall survival				Disease-free survival			
	HR	95% CI	Median OS Months (95% CI)	*p* Value	HR	95% CI	Median DFS Months (95% CI)	*p* Value
I-HGP + ND-HGP vs others	2.067	0.950–4.499	34 (25–58) 64 (44–107)	0.0672	1.775	0.883–3.565	14 (10–23) 25 (5–38)	0.1071
P-HGP + D-HGP vs others	0.905	0.380–2.157	64 (18–92) 44 (31–61)	0.8214	0.908	0.389–2.124	24 (2–52) 14 (10–25)	0.8246

*HR* Hazard ratio, *CI* Confidence interval, *OS* Overall survival, *DFS* Disease-free survival, *HGP* Histologic growth pattern, *I-HGP* Infiltrating HGP, *ND-HGP* Non-desmoplastic HGP, *P-HGP* Pushing HGP, *D-HGP* Desmoplastic HGP

By contrast, comparison of the P-HGP + D-HGP group with other profiles demonstrated no survival advantage, with HR close to 1 for both OS (HR, 0.905; *p* = 0.8214) and DFS (HR, 0.908; *p* = 0.8246). Finally, the analysis using four distinct groups based on all possible HGP combinations was not retained due to the limited sample size and statistical power.

In the univariate analysis of factors affecting survival, only PCI was significantly associated with OS, whereas DFS was influenced by PCI, MMR status, site of first metastasis, and use of preoperative chemotherapy (Supplementary File 2). In the multivariate analysis, none of these factors (including dominant HGP patterns) were significantly associated with OS or DFS (Supplementary File 3).

## Discussion

The main objective of this study was to evaluate any potential correlation between the HGP of PMCRC and LMCRC treated with curative intent. Using the same histopathologic evaluation protocol as defined in previous PMHGP studies and in LMHGP guidelines, we characterized the patterns and classified patients according to PM + LM HGP combinations.^23–26^ We reported a statistically significant correlation between PM and LM HGP, with a moderate strength of association (*p* = 0.0085; Φ = 0.3965). Despite some isolated cases of HGP discordance between nodules/slides, the consistency observed in most samples supports the reliability of dominant HGP classification in both PM and LM.

The secondary objective was to evaluate the prognostic value of PMCRC and LMCRC HGP. Our results demonstrated a nonsignificant trend toward worse outcomes for patients with the I-HGP + ND-HGP combination than for those with other HGP profiles, with a median OS of 34 versus 64 months (*p* = 0.0672), and a median DFS of 14 versus 25 months (*p* = 0.1071).

Moreover, the I-HGP + ND-HGP group was associated with a significantly higher PCI score, suggesting that more aggressive HGPs are associated with a higher peritoneal burden. However, multivariate analysis did not show any statistically significant associations.

Currently, HGP cannot be used as a biomarker to guide therapeutic decisions before liver resection for patients with LMCRC because its assessment requires analysis of the entire tumor-liver interface, which is not accessible on biopsy.^23^ Several studies are ongoing to evaluate dedicated imaging/radiomics tools and primary tumor characteristics that could predict LM HGP.^14,29,30^ Interestingly, this limitation could potentially be bypassed for patients presenting with both PM and LM. In this study, a significant correlation between HGP at both sites (*p* < 0.01) suggested that the HGP identified from peritoneal nodules may indirectly reflect the dominant hepatic pattern. This could be particularly valuable for prognostic estimation and refining of surgical decision-making.

During diagnostic staging laparoscopy, a limited number of peritoneal nodules can be safely resected for HGP analysis. As shown in this study and previously reported by Kamdem et al.^26^ inter-nodular variability within the same patient is minimal, supporting the idea that a few nodules may reliably represent the overall peritoneal disease. This strategy is especially relevant given that sampling the entire peritoneum is not feasible during exploratory laparoscopy. Identifying a poor-prognosis HGP (infiltrating-HGP) preoperatively could help the decision-making on the most appropriate treatment (preoperative chemotherapy vs upfront CRS).

The limitations of this study, such as its retrospective design, together with the small sample size, may reduce the generalizability of the results. The limited number of peritoneal and hepatic nodules analyzed per patient also could have influenced the representativeness of the observed HGP. In fact, given the small size of each HGP combination group, these results should be interpreted with caution because they remain statistically fragile and may be subject to a type 1 error. External validation in a larger cohort is therefore required. Moreover, the potential impact of the selected subset also should be considered because it may have enriched the cohort for specific biologic profiles.

In addition, the population was heterogeneous regarding the timing of metastasis onset, surgical management, and perioperative treatments. Some patients received preoperative and/or postoperative chemotherapy, with or without HIPEC, whereas others were chemo-naive at the time of surgery, which may have introduced selection bias. Given potential cofounders (PCI, chemotherapy, synchronicity), these conclusions regarding the prognostic value of PMCRC and LMCRC HGP should be framed as exploratory. The absence of statistical significance must be interpreted with caution considering the small size of the cohort and the high risk of type 2 error.

Altogether, these results warrant confirmation in larger, prospective, multicentric studies with a more homogeneous cohort to increase statistical power and better evaluate the importance of PM and LM HGP as independent prognostic factors. In perspective, a prospective pilot study incorporating standardized treatment protocols and allowing for longitudinal sampling via the analysis of PM collected during exploratory laparoscopy could enable early HGP determination before any treatment. A “theoretical” tailored decision based on several prognostic factors, including PM HGP, could be taken before any treatment and followed by the administration of “standard” treatment according to current recommendations at a multidisciplinary meeting. This should allow the prospective evaluation of the predictive value of HGP and other major predictive factors in the decision-making process for selecting patients who are good candidates for surgery.

## Conclusion

This study highlighted a significant correlation between HGP in PM and LM of CRC. Although survival analyses demonstrated non-significant trends, HGP assessment may help refine the indication for CRS and liver resection by anticipating disease aggressiveness and recurrence patterns. This finding reinforces the potential role of HGP as a prognostic biomarker and ultimately supports a shift toward individualized management in metastatic CRC by adapting the treatment strategy to each patient’s specific HGP profile.

## Supplementary Information

Below is the link to the electronic supplementary material.

Supplementary file1 (DOCX 24 KB)

Supplementary file2 (DOCX 25 KB)

Supplementary file3 (DOCX 17 KB)
