# Supplementary material for: Correlations Between the Histologic Growth Patterns of Peritoneal and Liver Metastases From Colorectal Cancer
Source: Ann Surg Oncol. 2026 Jun 29;33(12):10415–24. doi: 10.1245/s10434-026-20005-8 (PMC13675321; doi:10.1245/s10434-026-20005-8)
Supplement: Supplementary file 1 [file 10434_2026_20005_MOESM1_ESM.docx]

**Supplementary file 1.** Clinico-pathological characteristics of the population by center (Jules Bordet Institute and Cliniques Universitaires Saint-Luc)

| **Variables** | **Population**  (N=44) | **Jules Bordet Institute**  (N=26) | **Cliniques Universitaires**  **Saint-Luc**  (N=18) | **p-value** |
| --- | --- | --- | --- | --- |
| **Sex, n (%)** |  |  |  | 0.2250 |
| M | 17 (38.6%) | 8 (30.8%) | 9 (50.0%) |  |
| F | 27 (61.4%) | 18 (69.2%) | 9 (50.0%) |  |
| **Age, n (%)** |  |  |  | 0.7597 |
| ≤ 58 | 21 (47.7%) | 12 (46.2%) | 9 (50.0%) |  |
| > 58 | 23 (52.3%) | 14 (53.8%) | 9 (50.0%) |  |
| **ASA, n (%)** |  |  |  | 0.2317 |
| 1-2 | 31 (70.5%) | 20 (76.9%) | 11 (61.1%) |  |
| 3 | 13 (29.5%) | 6 (23.1%) | 7 (38.9%) |  |
| **Site, n (%)** |  |  |  | 0.6337 |
| Colon | 40 (90.9%) | 23 (88.5%) | 17 (94.4%) |  |
| Rectum | 4 (9.1%) | 3 (11.5%) | 1 (5.6%) |  |
| **T, n (%)** |  |  |  | 0.3586 |
| 3 | 19 (43.2%) | 13 (50.0%) | 6 (33.3%) |  |
| 4 | 25 (56.8%) | 13 (50.0%) | 12 (66.7%) |  |
| **N, n (%)** |  |  |  | 0.8488 |
| 0 | 8 (18.2%) | 4 (15.4%) | 4 (22.2%) |  |
| 1 | 15 (34.1%) | 9 (34.6%) | 6 (33.4%) |  |
| 2 | 21 (47.7%) | 13 (50.0%) | 8 (44.4%) |  |
| **Grade, n (%)** |  |  |  | 0.8597 |
| Low grade | 9 (20.5%) | 6 (23.1%) | 3 (16.7%) |  |
| Moderate | 33 (75.0%) | 19 (73.1%) | 14 (77.7%) |  |
| High grade | 2 (4.5%) | 1 (3.8%) | 1 (5.6%) |  |
| **Mucinous, n (%)** |  |  |  | 0.6817 |
| No | 37 (84.1%) | 21 (80.8%) | 16 (88.9%) |  |
| Yes | 7 (15.9%) | 5 (19.2%) | 2 (11.1%) |  |
| **MMR, n (%)** *– missing: 7* |  |  |  | 1.0000 |
| MSS | 36 (97.3%) | 22 (95.7%) | 14 (100.0%) |  |
| MSI | 1 (2.7%) | 1 (4.3%) | 0 (0.0%) |  |
| **KRAS, n (%)** *– missing: 12* |  |  |  | 0.7178 |
| Wild | 14 (43.8%) | 8 (40.0%) | 6 (50.0%) |  |
| Mutated | 18 (56.2%) | 12 (60.0%) | 6 (50.0%) |  |
| **BRAF, n (%)** *– missing: 19* |  |  |  | 1.0000 |
| Wild | 24 (96.0%) | 16 (64.0%) | 8 (32.0%) |  |
| Mutated | 1 (4.0%) | 1 (4.0%) | 0 (0.0%) |  |
| **Preoperative chemo, n (%)** |  |  |  | 0.0879 |
| Before both | 19 (43.2%) | 8 (30.8%) | 11 (61.1%) |  |
| Only before CRS | 5 (11.3%) | 3 (11.5%) | 2 (11.1%) |  |
| Only before LM resection | 8 (18.2%) | 7 (26.9%) | 1 (5.6%) |  |
| None | 12 (27.3%) | 8 (30.8%) | 4 (22.2%) |  |

ASA: American Society of Anesthesiologists; CRS: cytoreductive surgery; D-HGP: desmoplastic HGP; HGP: histological growth pattern; I-HGP: infiltrating HGP; LM: liver metastases; MMR: mismatch repair; MSI: microsatellite instability; MSS: microsatellite stable; ND-HGP: non-desmoplastic HGP; P-HGP: pushing HGP.

**Supplementary file 1** (continued)

|  | **Variables** | **Population**  (N=44) | **Jules Bordet Institute**  (N=26) | **Cliniques Universitaires**  **Saint-Luc**  (N=18) | **p-value** |
| --- | --- | --- | --- | --- | --- |
| **PM** | **Chronology of PM, n (%)** |  |  |  | 0.2215 |
|  | Synchronous carcinosis | 19 (43.2%) | 9 (34.6%) | 10 (55.6%) |  |
|  | Metachronous carcinosis | 25 (56.8%) | 17 (65.4%) | 8 (44.4%) |  |
|  | **PCI, n (%)** |  |  |  | 0.5354 |
|  | ≤ 9.5 | 26 (59.1%) | 14 (53.8%) | 12 (66.7%) |  |
|  | > 9.5 | 18 (40.9%) | 12 (46.2%) | 6 (33.3%) |  |
|  | **CEA (ng/mL), n (%)** *– missing: 4* |  |  |  | 0.7501 |
|  | ≤ 5 | 24 (60.0%) | 15 (62.5%) | 9 (56.3%) |  |
|  | > 5 | 16 (40.0%) | 9 (37.5%) | 7 (43.8%) |  |
|  | **Completeness of CRS, n (%)** |  |  |  | / |
|  | CC-0 | 44 (100.0%) | 26 (100.0%) | 18 (100.0%) |  |
|  | **HIPEC, n (%)** |  |  |  | 0.1607 |
|  | No | 10 (22.7%) | 8 (30.8%) | 2 (11.1%) |  |
|  | Yes | 34 (77.3%) | 18 (69.2%) | 16 (88.9%) |  |
|  | **Nb of peritoneal nodules analyzed** |  |  |  | 0.4526 |
|  | Mean ± SD | 1.9 ± 0.78 | 1.8 ± 0.75 | 2.0 ± 0.84 |  |
|  | **Nodule size (cm)** |  |  |  | / |
|  | Mean ± SD | 1.6 ± 0.83 | 1.6 ± 0.91 | 1.6 ± 0.69 |  |
|  | **PM HGP, n (%)** |  |  |  | 0.1037 |
|  | P-HGP ≥51% | 14 (31.8%) | 11 (42.3%) | 3 (16.7%) |  |
|  | I-HGP ≥51% | 30 (68.2%) | 15 (57.7%) | 15 (83.3%) |  |
| **LM** | **Chronology of LM, n (%)** |  |  |  | 0.5467 |
|  | Synchronous | 24 (54.5%) | 13 (50.0%) | 11 (61.1%) |  |
|  | Metachronous | 20 (45.5%) | 13 (50.0%) | 7 (38.9%) |  |
|  | **Nb of LM on preop imaging** |  |  |  | 0.4174 |
|  | Mean ± SD | 2.4 ± 1.59 | 2.6 ± 1.79 | 2.1± 1.21 |  |
|  | **Nb of LM analyzed** |  |  |  | 0.1384 |
|  | Mean ± SD | 1.4 ± 0.73 | 1.3 ± 0.60 | 1.6 ± 0.85 |  |
|  | **Liver metastases HGP, n (%)** |  |  |  | 1.0000 |
|  | D-HGP ≥51% | 16 (36.4%) | 9 (34.6%) | 7 (38.9%) |  |
|  | ND-HGP ≥51% | 28 (63.6%) | 17 (65.4%) | 11 (61.1%) |  |
| **PM + LM** | **First metastasis treated with curative intent, n (%)** |  |  |  | 0.4366 |
|  | Peritoneum | 15 (34.1%) | 7 (26.9%) | 8 (44.4%) |  |
|  | Liver | 16 (36.4%) | 11 (42.3%) | 5 (27.8%) |  |
|  | Peritoneum + Liver | 13 (29.5%) | 8 (30.8%) | 5 (27.8%) |  |

CC: completeness of cytoreduction; CRS: cytoreductive surgery; D-HGP: desmoplastic HGP; HGP: histological growth pattern; HIPEC: hyperthermic intraperitoneal chemotherapy; I-HGP: infiltrating HGP; LM: liver metastases; ND-HGP: non-desmoplastic HGP; P-HGP: pushing HGP; PCI: peritoneal cancer index; PM: peritoneal metastases; SD: standard deviation.
