# Supplementary material for: Correlations Between the Histologic Growth Patterns of Peritoneal and Liver Metastases From Colorectal Cancer
Source: Ann Surg Oncol. 2026 Jun 29;33(12):10415–24. doi: 10.1245/s10434-026-20005-8 (PMC13675321; doi:10.1245/s10434-026-20005-8)
Supplement: Supplementary file 2 [file 10434_2026_20005_MOESM2_ESM.docx]

**Supplementary file 2.** Univariate analysis of clinical, demographic, and histological variables on outcomes

| **Variables** | **Population**  (N=37) | **Overall survival** | | **Disease-free survival** | |
| --- | --- | --- | --- | --- | --- |
|  |  | Median OS  (95% CI, in months) | p-value | Median DFS  (95% CI, in months) | p-value |
| **Sex, n (%)** |  |  | 0.353 |  | 0.655 |
| M | 15 (40.5%) | 55 (29-107) |  | 23 (4-29) |  |
| F | 22 (59.5%) | 44 (25-67) |  | 15 (7-29) |  |
| **Age, n (%)** |  |  | 0.570 |  | 0.829 |
| ≤ 58 | 17 (45.9%) | 52 (29-107) |  | 14 (5-36) |  |
| > 58 | 20 (54.1%) | 44 (25-65) |  | 16 (7-29) |  |
| **ASA, n (%)** |  |  | 0.484 |  | 0.912 |
| 1-2 | 24 (64.9%) | 55 (34-88) |  | 17 (7-29) |  |
| 3 | 13 (35.1%) | 41 (24-75) |  | 14 (4-29) |  |
| **Site, n (%)** |  |  | 0.943 |  | 0.968 |
| Colon | 34 (91.9%) | 44 (30-67) |  | 15 (10-25) |  |
| Rectum | 3 (8.1%) | 55 (34-NA) |  | 28 (12-NA) |  |
| **T, n (%)** |  |  | 0.918 |  | 0.685 |
| 3 | 14 (37.8%) | 55 (25-92) |  | 15 (7-29) |  |
| 4 | 23 (62.2%) | 43 (20-67) |  | 17 (7-29) |  |
| **N, n (%)** |  |  | 0.383 |  | 0.368 |
| 0 | 7 (18.9%) | 44 (23-NA) |  | 12 (4-29) |  |
| 1 | 12 (32.4%) | 66 (20-NA) |  | 19 (2-56) |  |
| 2 | 18 (48.7%) | 44 (29-92) |  | 15 (7-36) |  |
| **Grade, n (%)** |  |  | 0.397 |  | 0.105 |
| Low grade | 7 (18.9%) | 41 (20-64) |  | 6 (0.6-28) |  |
| Moderate | 29 (78.4%) | 55 (31-88) |  | 18 (12-31) |  |
| High grade | 1 (2.7%) | - |  | - |  |
| **Mucinous, n (%)** |  |  | 0.579 |  | 0.983 |
| No | 30 (81.1%) | 44 (30-67) |  | 16 (10-29) |  |
| Yes | 7 (18.9%) | 58 (24-NA) |  | 14 (4-25) |  |
| **MMR, n (%)** *– missing: 7* |  |  | 0.901 |  | **0.015** |
| MSS | 31 (96.9%) | 52 (31-67) |  | 16 (12-29) |  |
| MSI | 1 (3.1%) | 64 (NA) |  | 4 (NA) |  |
| **KRAS, n (%)** *– missing: 12* |  |  | 0.691 |  | 0.566 |
| Wild | 11 (39.3%) | 52 (29-NA) |  | 16 (4-29) |  |
| Mutated | 17 (60.7%) | 58 (25-92) |  | 15 (7-52) |  |
| **Preoperative chemo, n(%)** |  |  | 0.059 |  | **0.016** |
| Before both | 18 (48.6%) | 34 (25-67) |  | 13 (6-19) |  |
| Only before CRS | 4 (10.8%) | - |  | 67 (4-NA) |  |
| Only before LM resection | 6 (16.3%) | 92 (24-NA) |  | 37 (10-NA) |  |
| None | 9 (24.3%) | 55 (18-64) |  | 14 (4-29) |  |

ASA: American Society of Anesthesiologists; CI: confidence interval; CRS: cytoreductive surgery; D-HGP: desmoplastic HGP; DFS: disease-free survival; HGP: histological growth pattern; I-HGP: infiltrating HGP; LM: liver metastases; MMR: mismatch repair; MSI: microsatellite instability; MSS: microsatellite stable; ND-HGP: non-desmoplastic HGP; OS: overall survival; P-HGP: pushing HGP.

**Supplementary file 2** (continued)

|  | **Variables** | **Population**  (N=37) | **Overall survival** | | **Disease-free survival** | |
| --- | --- | --- | --- | --- | --- | --- |
|  |  |  | Median OS  (95% CI, in months) | p-value | Median DFS  (95% CI, in months) | p-value |
| **PM** | **Chronology of PM, n (%)** |  |  | 0.176 |  | 0.654 |
|  | Synchronous carcinosis | 17 (45.9%) | 34 (24-67) |  | 16 (10-28) |  |
|  | Metachronous carcinosis | 20 (54.1%) | 58 (30-92) |  | 16 (5-36) |  |
|  | **PCI, n (%)** |  |  | **0.019** |  | **0.003** |
|  | ≤ 9.5 | 23 (62.2%) | 64 (29-107) |  | 23 (12-38) |  |
|  | > 9.5 | 14 (37.8%) | 36 (25-52) |  | 11 (4-15) |  |
|  | **CEA (ng/mL), n (%)** *– missing: 4* |  |  | 0.166 |  | 0.193 |
|  | ≤ 5 | 19 (57.6%) | 64 (41-88) |  | 22 (10-52) |  |
|  | > 5 | 14 (42.4%) | 36 (24-61) |  | 15 (5-31) |  |
|  | **Completeness of CRS, n (%)** |  |  | - |  | - |
|  | CC-0 | 37 (100%) | 52 (33-64) |  | 16 (12-28) |  |
|  | **HIPEC, n (%)** |  |  | 0.645 |  | 0.945 |
|  | No | 8 (21.6%) | 52 (17-NA) |  | 13 (2-NA) |  |
|  | Yes | 29 (78.4%) | 44 (33-64) |  | 16 (12-28) |  |
|  | **Nodule size (cm)** |  |  | 0.185 |  | 0.134 |
|  | ≤ 1.6 | 29 (78.4%) | 41 (30-67) |  | 15 (10-23) |  |
|  | > 1.6 | 8 (21.6%) | 58 (17-NA) |  | 33 (2-NA) |  |
|  | **PM HGP, n (%)** |  |  | 0.495 |  | 0.330 |
|  | P-HGP ≥51% | 12 (32.4%) | 64 (24-107) |  | 27 (4-52) |  |
|  | I-HGP ≥51% | 25 (67.6%) | 41 (30-58) |  | 14 (10-25) |  |
| **LM** | **Chronology of LM, n (%)** |  |  | 0.301 |  | 0.289 |
|  | Synchronous | 21 (56.8%) | 61 (30-92) |  | 19 (12-32) |  |
|  | Metachronous | 16 (43.2%) | 44 (29-64) |  | 13 (4-25) |  |
|  | **Nb of LM on preop imaging** |  |  | 0.428 |  | 0.178 |
|  | ≤ 2 | 31 (83.8%) | 52 (31-67) |  | 14 (7-23) |  |
|  | > 2 | 6 (16.2%) | 48 (25-NA) |  | 29 (10-NA) |  |
|  | **Liver metastases HGP, n (%)** |  |  | 0.181 |  | 0.399 |
|  | D-HGP ≥51% | 14 (37.8%) | 64 (30-107) |  | 23 (4-52) |  |
|  | ND-HGP ≥51% | 23 (62.2%) | 36 (29-61) |  | 14 (10-23) |  |
| **PM + LM** | **First metastasis treated with curative intent, n (%)** |  |  | 0.178 |  | **0.020** |
|  | Peritoneum | 13 (35.1%) | 41 (29-88) |  | 14 (7-25) |  |
|  | Liver | 13 (35.1%) | 61 (25-130) |  | 32 (14-63) |  |
|  | Peritoneum + Liver | 11 (29.8%) | 43 (20-67) |  | 6 (2-22) |  |

CC: completeness of cytoreduction; CI: confidence interval; CRS: cytoreductive surgery; D-HGP: desmoplastic HGP; DFS: disease-free survival; HGP: histological growth pattern; HIPEC: hyperthermic intraperitoneal chemotherapy; I-HGP: infiltrating HGP; LM: liver metastases; ND-HGP: non-desmoplastic HGP; OS: overall survival; P-HGP: pushing HGP; PCI: peritoneal cancer index; PM: peritoneal metastases; SD: standard deviation.
