# Supplementary material for: Correlations Between the Histologic Growth Patterns of Peritoneal and Liver Metastases From Colorectal Cancer
Source: Ann Surg Oncol. 2026 Jun 29;33(12):10415–24. doi: 10.1245/s10434-026-20005-8 (PMC13675321; doi:10.1245/s10434-026-20005-8)
Supplement: Supplementary file 3 [file 10434_2026_20005_MOESM3_ESM.docx]

**Supplementary file 3.** Overall survival and disease-free survival on multivariate analysis (Cox regression model)

|  | **Variables** | **Overall survival** | | **Disease-free survival** | |
| --- | --- | --- | --- | --- | --- |
|  |  | HR (95% CI) | p-value | HR (95% CI) | p-value |
| **Overall population**  (N=37) | PCI > 9.5 | 2.20 (0.69-7.09) | 0.1845 | 2.43 (0.87-6.80) | 0.0909 |
|  | CEA > 5 ng/mL | 1.86 (0.79-4.39) | 0.1586 | 1.68 (0.78-3.64) | 0.1851 |
|  | I-HGP ≥51% | 0.79 (0.27-2.30) | 0.6680 | 0.93 (0.38-2.32) | 0.8834 |
|  | ND-HGP ≥51% | 1.66 (0.62-4.48) | 0.3165 | 1.30 (0.54-3.11) | 0.5583 |

CI: confidence interval; HGP: histological growth pattern; HR: hazard ratio; I-HGP: infiltrating HGP; ND-HGP: non-desmoplastic HGP; PCI: peritoneal cancer index.
